# Purely organic room-temperature phosphorescence sensitizers for OLEDs

**DOI:** 10.1039/d6sc03065c

**Published:** 2026-06-12

**Authors:** Hao Liu, Yan Fu, Jacky W. Y. Lam, Ben Zhong Tang, Zujin Zhao

**Affiliations:** a State Key Laboratory of Luminescent Materials and Devices, Guangdong Provincial Key Laboratory of Luminescence from Molecular Aggregates, South China University of Technology Guangzhou 510640 China mszjzhao@scut.edu.cn; b Guangdong Basic Research Center of Excellence for Aggregate Science, School of Science and Engineering, The Chinese University of Hong Kong (Shenzhen) Longgang, Shenzhen Guangdong 518172 China; c Department of Chemistry and the Hong Kong Branch of Chinese National Engineering Research Center for Tissue Restoration and Reconstruction, The Hong Kong University of Science and Technology Clear Water Bay Kowloon Hong Kong 999077 China

## Abstract

Organometallic phosphorescent materials have been developed as critical luminescent materials for organic light-emitting diodes (OLEDs). However, most purely organic room-temperature phosphorescence (RTP) materials without any heavy metals still lack competitiveness for use in OLEDs. Recently, significant progress has been made regarding purely organic RTP materials, and their electroluminescence (EL) performances have become comparable to those of traditional phosphorescent complexes. In this perspective, advancements and proposed design strategies relating to efficient purely organic RTP materials are summarized. Furthermore, the promising application of these RTP materials as sensitizers for narrow-spectrum multi-resonance emitters is also discussed and an outlook is provided, which is conducive to the development of high-resolution OLEDs. It is expected that this perspective will provide valuable guidelines for advancing robust purely organic RTP sensitizers and further promoting the OLED industry.

## Abstract 2

Purely organic room-temperature phosphorescence materials are significant as sensitizers in OLEDs, facilitating next-generation displays. In this perspective, the progress and design principles relating to effective RTP sensitizers are summarized.

## Graphical abstract

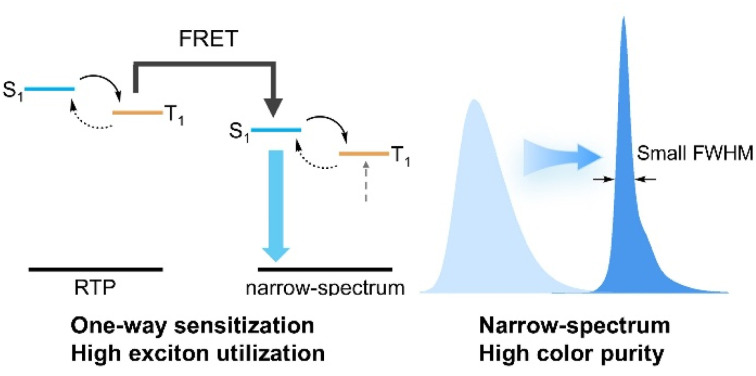

## Introduction

Organic luminescent materials have been developed for over half a century, and they have played a crucial role in organic light-emitting diodes (OLEDs),^1,2^ secure communications,^3^ encryption,^4,5^ bioimaging,^6,7^*etc.* Based on the spin statistics of electrons, electro-generated excitons consist 75% of triplet states and 25% of singlet states, where the triplet states are kinetically hindered from converting to singlet states or ground states.^8^ One of the most successful strategies for utilizing triplet excitons is phosphorescent organometallic complexes, which generally incorporate heavy metals (*e.g.*, Ir or Pt) to enhance spin–orbit coupling (SOC).^9,10^ They can achieve nearly 100% exciton utilization efficiency (EUE), and their extraordinary performance has attracted considerable interest across multiple fields.^11,12^ The most successful application of these phosphorescent materials is in OLEDs, which have taken a large share of the mobile display market.^13^ Nevertheless, the presence of expensive heavy metals brings about challenges in controlling the cost of OLEDs, necessitating the development of low-cost purely organic luminescent materials.

Recently, several types of high-EUE purely organic luminescent materials have been proposed, featuring distinct mechanisms, including room-temperature phosphorescence (RTP),^14^ thermally activated delayed fluorescence (TADF),^15^ hybrid local and charge-transfer (HLCT),^16^ and organic radicals.^17^ TADF materials typically consist of electron donor (D) and acceptor (A) moieties, wherein the D–A structure can induce intramolecular charge transfer (ICT) characteristics.^18^ Reverse intersystem crossing (RISC) facilitates the conversion of excitons from the lowest triplet (T_1_) state to the lowest singlet (S_1_) state, enabling nearly 100% EUE and impressive electroluminescence (EL) performance, exceeding 30%.^19–22^ This TADF characteristic was also discovered in polycyclic aromatic compounds constructed by alternating heteroatoms with opposite electronegativities, termed as multi-resonance (MR) TADF materials.^23–25^ Such MR-TADF materials can realize narrow-spectrum emission, and they are regarded as ideal terminal emitters for high-definition displays.^26–28^ However, the rates of RISC (*k*_RISC_s) of MR-TADF molecules remain relatively low due to the insufficient separation of the frontier molecular orbitals, which results in a large energy gap (Δ*E*_ST_) between S_1_ and T_1_ states.^29^ Thus, sensitization strategies were proposed to enhance the EL efficiency and operational stability of MR-TADF emitters, where TADF or metallic phosphorescent materials are employed as sensitizers.^30–34^ However, possible repeated cycles between the S_1_ and T_1_ states of TADF materials may lead to prolonged exciton lifetimes and inevitable non-radiative loss.^35^ Conversely, purely organic RTP emitters are potentially applicable in sensitized OLEDs due to their high EUEs. Notably, the unidirectional radiative decay characteristics of RTP materials minimize the spin-flip cycles between triplet and singlet states, which is favourable for increasing the efficiency and stability of devices. Recently, some RTP materials have been applied to sensitized OLEDs that utilize narrow-spectrum fluorescent materials as terminal emitters.^36,37^ Meanwhile, purely organic RTP materials contain no expensive metals, which can effectively reduce the cost of materials and thus facilitate application in OLED panels.

Although purely organic RTP materials offer several advantages over TADF materials, the realization of efficient phosphorescent emission from them remains challenging. Over the past decade, various strategies for enhancing RTP in organic materials have been proposed.^38–40^ However, numerous RTP materials can only function efficiently under specific conditions. For example, crystallization is a commonly used method to rigidify organic molecules and stabilize triplet states.^41^ In OLED fabrication, vacuum evaporation and solution-processing technologies are widely used, whereby amorphous films are utilized as the emissive layers.^1,42^ Moreover, to ensure the reproducibility and stability of OLEDs, the use of additives to facilitate RTP emission should be avoided.^43^ As a result, several conventional purely organic RTP emitters only provided low EUEs in OLEDs,^44,45^ which could be attributed to inefficient ISC and slow phosphorescent decay rates. Therefore, the application of purely organic RTP materials in OLEDs still faces several obstacles.

To date, research on purely organic RTP materials has mainly focused on molecular design strategies and the photophysical properties, and the scarcity of reports on their EL performance has impeded the progress of RTP-based OLEDs. Herein, we review recent advancements in purely organic RTP materials and their corresponding EL performances. Effective strategies to facilitate RTP emission are summarized, and the progress of the EL performances of these RTP emitters is also discussed. More importantly, the key factors governing high EL performances in RTP materials and their potential application as purely organic sensitizers are systematically analysed and discussed, with prospective development paths proposed.

## Design principles of RTP materials

### Photophysical processes in RTP materials

**Fig. 1:**
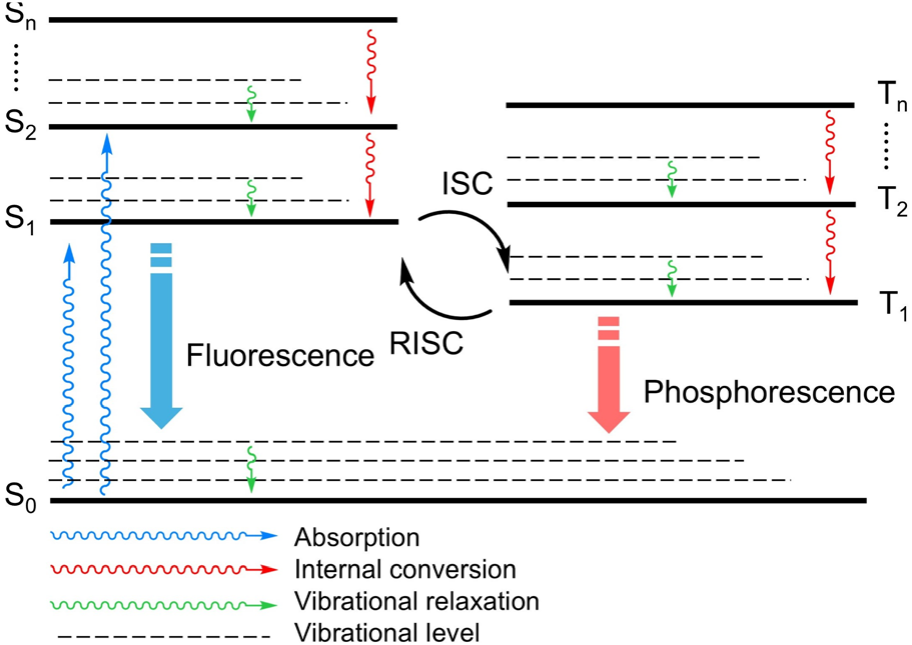
The Jablonski diagram for photophysical processes.

The Jablonski diagram is widely used to understand the generation and deactivation pathways of singlet and triplet excitons (Fig. 1). Under photoexcitation, normally, only singlet excitons are generated according to the principle of spin conservation. Thus, triplet excitons can only be generated through ISC from the singlet manifold. The excitons in higher-lying singlet or triplet states will rapidly relax to the lowest S_1_ and T_1_ states *via* internal conversion (IC) processes. Besides, ISC from S_1_ to higher-level triplet states followed by IC can also effectively populate the T_1_ states.

Notably, the T_1_ state is inherently susceptible to deactivation through various competitive pathways. Beyond internal non-radiative decay, such as vibrational dissipation^46^ and RISC, and triplet–triplet annihilation (TTA) and singlet–triplet annihilation (STA),^47^ triplet excitons in purely organic systems are highly sensitive to quenching by molecular oxygen (^3^O_2_) under ambient conditions.^48^ This process is governed by triplet–triplet energy transfer, wherein interaction between the T_1_ state of the luminophore and the triplet ground state of oxygen leads to the formation of singlet oxygen and the non-radiative deactivation of the molecule. Consequently, achieving efficient RTP requires synergistic effects: employing rigid matrices or crystalline packing to provide an effective oxygen barrier and minimize non-radiative loss, while enhancing the SOC constant to achieve a high radiative rate (*k*_P_) and ISC rate (*k*_ISC_), both of which are essential for maximizing photoluminescence quantum yields (*Φ*_PL_s). Therefore, the design principles for efficient RTP materials primarily involve two aspects. Firstly, optimizing the morphology and host matrix to suppress non-radiative decay and modulate intermolecular interactions. Secondly, enhancing SOC to facilitate spin-flip processes between singlet and triplet states, as well as between triplet and ground states.

### Morphology dependence of RTP materials

#### Crystallization

**Fig. 2:**
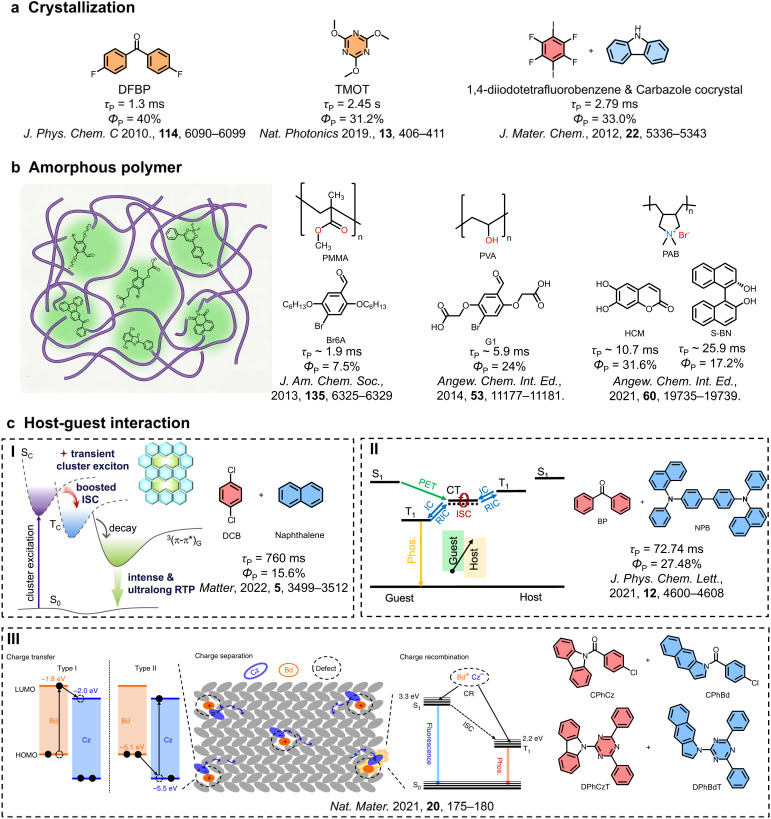
Examples of the morphology dependence of RTP materials: (a) crystallization-induced phosphorescence; (b) RTP in amorphous polymer matrices; and (c) host–guest interaction and its mechanisms, where the red molecules represent the host phase and blue molecules are dopants: (I) phosphorescence decay *via* intermolecular orbitals, reproduced with permission: copyright 2022, Elsevier;^60^ (II) photoinduced electron transfer, reproduced with permission: copyright 2021, American Chemical Society;^62^ and (III) charge-separated-state-induced afterglow, reproduced with permission: copyright 2021, Springer-Nature.^61^

Many RTP phenomena were observed in aqueous solutions during the early stages of development. Although phosphorescence in anthracene crystals was reported in the 1960s,^49^ its weak intensity under ambient conditions limited its practical application. A rigid polymer matrix can effectively restrict molecular motion, facilitating phosphorescent emission. Ghiggino *et al.* observed the phosphorescent emission of tryptophan by doping it into a rigid polyvinyl-alcohol (PVA) matrix.^50^ Levy *et al.* processed dye-doped sol–gel silica glasses, where RTP phenomena were detected.^51^ In 2010, Tang *et al.* reported a series of halogenated benzophenone derivatives that could only present strong phosphorescence in single crystals, namely crystal-induced phosphorescence (Fig. 2).^41^ Even though the carbonyl group and halogens are favoured to increase SOC constants, molecular rotations in solutions or amorphous films induce severe exciton quenching. Crystallization effectively restricts intramolecular motion through an extensive network of hydrogen bonds. Consequently, 4,4′-difluorobenzophenone (DFBP) molecules achieved persistent phosphorescence with a long lifetime (*τ*_P_) of 1.3 ms and a high phosphorescent quantum yield (*Φ*_P_) of 40%. Huang *et al.* designed 2,4,6-trimethoxy-1,3,5-triazin (TMOT) derivatives and proposed H-aggregation-assisted ultra-long phosphorescent emission.^52^ The dimeric states of H-aggregates can effectively stabilize triplet states of TMOT, bringing about a long *τ*_P_ of 2.45 s and a good *Φ*_P_ of 31.2%. In addition, several RTP phenomena were observed in organic co-crystals. For example, Jin *et al.* adopted the halogen-rich compound 1,4-diiodotetrafluorobenzene and carbazole to grow co-crystalline structures, which utilized C–I⋯π interactions to promote phosphorescent emission. A suspension of microparticles of co-crystal exhibited *τ*_P_ of 2.79 ms and *Φ*_P_ of 33.0%.^53^

#### Amorphous polymers

Polymerization is an effective method to increase inter-/intra-molecular interactions, where the stabilization of the environment is favourable for RTP emission. Fig. 2b displays some examples of RTP emission in amorphous polymer matrices. Poly(methyl methacrylate) (PMMA) is a widely used host matrix for the encapsulation of RTP materials,^54,55^ where the glassy state provides a rigid microenvironment. For hydrophilic emitters, PVA is a more appropriate selection, where strong hydrogen bonding can be established.^56^ In addition, other kinds of polymers, such as polyacrylamide^57^ and polyamide 6,^58^ are also RTP matrix candidates. Ionic bonding polymers are emerging as ideal matrices for long-lived RTP emission, as the strong electrostatic interactions between opposite ions can greatly stabilize triplet excitons. Ma *et al.* developed a series of new RTP systems by doping fluorescent dyes into ionic polymers of poly-diallyldimethylammonium bromide (PAB) and poly-diallyldimethylammonium chloride (PAC), where the presence of bromine and chlorine ions provided both structural rigidity and external heavy-atom effects.^59^ The rigid polymer PAB can trigger RTP emission from various organic dyes.

#### Host–guest interaction

Doping emitters into specific host materials can induce excess intermolecular interactions and modulate excited states, as well as avoid concentration quenching. Naphthalene is a classic aromatic fluorescent dye, which possesses negligible phosphorescence at room temperature. Even upon crystallization, naphthalene still suffers from slow ISC and exciton quenching. Tang *et al.* employed halogenated benzene as a guest dopant in naphthalene, successfully inducing RTP features,^60^ and the mixture exhibited *τ*_P_ of 760 ms and *Φ*_P_ of 15.6% in a 1,4-dichlorobenzene (DCB) host, in which the delocalized molecular orbitals provided a new decay path for triplet excitons, and considerable SOC constants were realized between several singlet and triplet states, facilitating RTP emission (Fig. 2c). In 2021, Liu *et al.* reported a unique phenomenon in which the carbazole isomer 1*H*-benzo[*f*]indole (Bd) in commercially bought carbazole raw materials could induce strong RTP in some molecules.^61^ Trace Bd inside (9*H*-carbazol-9-yl)(4-chlorophenyl)methanone (CphCz) and 4,6-diphenyl-2-carbazolyl-1,3,5-triazine (DphCzT) could trigger ultra-long RTP emission, where the energy level difference between Bd and carbazole could form charge-separated states under excitation, and radical traps were formed on Bd molecules, thus elongating the exciton recombination time (Fig. 2c). Su *et al.* doped a series of biphenyl derivatives with different substitutions into a benzophenone host, realizing ultra-long RTP emission.^62^ Photoinduced electron transfer (PET) can lead to an intermediate CT state that can go through an efficient ISC process (Fig. 2c). Finally, through energy transfer between the PET state and triplet states of the guests, a high *Φ*_P_ of 27.48% with *τ*_P_ of 72.74 ms was realized for a combination of benzophenone (BP) host and 4,4′-bis[*N*-(1-naphthyl)-*N*-phenylamino]biphenyl (NPB) dopant.

#### Enhancing spin-flipping of excitons

As illustrated in the Jablonski diagram, one of the key procedures during phosphorescent emission is the spin-flipping of excitons. Efficient ISC and radiative decay of triplet excitons are significant for RTP emission. ISC can effectively accumulate triplet excitons, which determines the upper limit of *Φ*_P_. The ISC rate (*k*_ISC_) can be expressed as:^63^
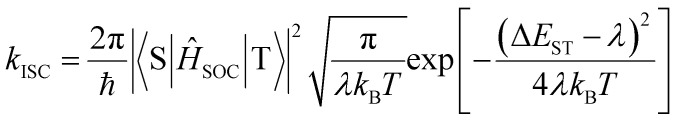
where ℏ denotes the reduced Planck's constant, S|*Ĥ*_SOC_|T represents the SOC matrix between triplet and singlet states, *λ* is the total reorganization energy, *k*_B_ is the Boltzmann constant, *T* is the temperature, and Δ*E*_ST_ is the energy gap between singlet and triplet states. According to this formula, enhancing the SOC matrix constant and reducing Δ*E*_ST_ are regarded as two important factors to facilitate *k*_ISC_.

**Fig. 3:**
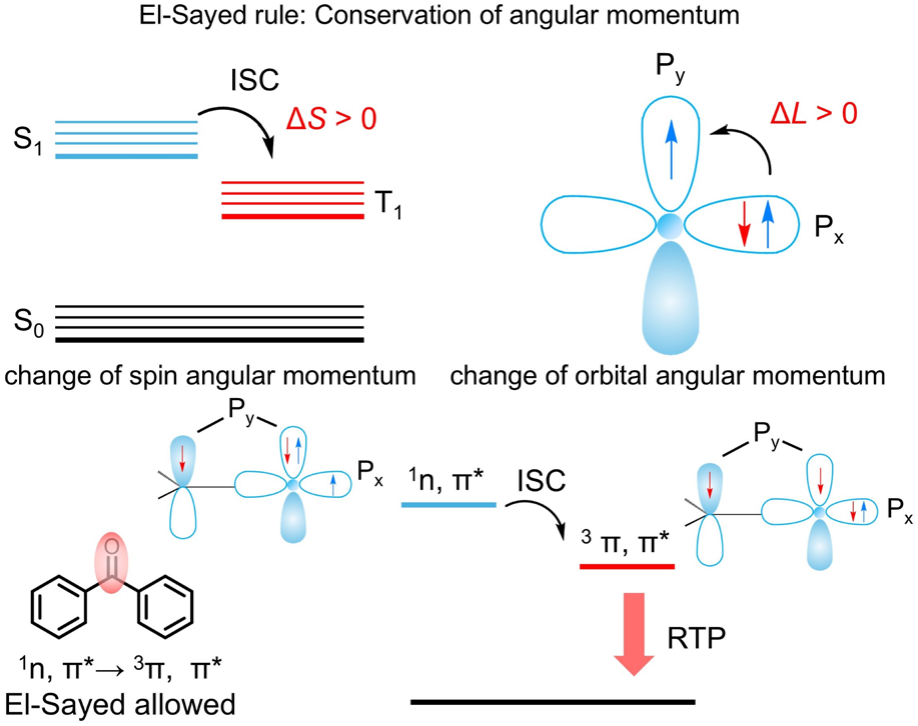
A schematic diagram of the El-Sayed rule.

One important approach towards large SOC is introducing lone-pair electrons.^64,65^ According to the El-Sayed rule, the conservation of angular momentum is pivotal to the spin-flip process.^66^ SOC is significantly enhanced between the excited states with different orbital configurations (*e.g.*, ^1^n, π* and ^3^π, π*) compared with the states having similar characters, because the change in spin angular momentum can be compensated by a corresponding change in orbital angular momentum (Fig. 3). The engagements of atoms containing lone-pair electrons (*e.g.* O, N, and S) can modulate the excited states of both singlet and triplet states, providing (n, π*) transitions. The SOC matrix constants between (n, π*) and (π, π*) are generally much larger, which are sufficient for the ISC process. Practically, a theoretical calculation can evaluate the SOC matrix constants between different states. The SOC matrix constant is structurally dependent, and the optimized molecular structures should be output first *via* commercialized software (*e.g.* Gaussian or ORCA). After obtaining the optimal structures, the SOC matrix constant can further be calculated by specific software (*e.g.* ORCA or Dalton). Generally, the same density functional and basis set are consistently employed for both geometry optimization and SOC calculations. Regarding the selection of functionals, initial screening can be performed based on the molecular structure. Specifically, D–A type RTP emitters dominated by charge transfer transitions prefer functionals with a high Hartree–Fock exchange fraction (*e.g.*, M06-2X, CAM-B3LYP, and wB97X-D). Conversely, conventional RTP emitters dominated by localized excitation may use functionals with a low Hartree–Fock exchange fraction (*e.g.*, B3LYP and PBE0). As for the basis set, the Pople and def2 basis sets are commonly selected. To ensure computational accuracy, the 6-31G**, def2-SVP, or def-TZVP level is typically recommended at least. For molecules containing relatively few atoms, the higher-accuracy def2-TZVP basis set can also be considered. For example, Yuan *et al.* reported two D–A type molecules, *m*-PBCM and *p*-PBCM, consisting of carbazole and carbonyl groups, which exhibited persistent RTP emission on the sub-second scale.^67^ The calculated SOC matrix constants between the S_1_ and T_3_ states were 1.97 and 2.18 cm^−1^ for *m*-PBCM and *p*-PBCM, respectively, offering high potential for ISC.

In addition, the heavy-atom effect is another effective method to augment SOC, as the coupling strength scales with *Z*^4^, where *Z* represents the nuclear charge of an atom.^68^ Commercialized phosphorescent materials contain heavy metals, where the engagement of outer electron orbitals can provide sufficient spin angular momentum. For purely organic emitters, the incorporation of heavy atoms is mainly concentrated on halogens or chalcogens. For instance, He *et al.* added halogens at the end of the alkyl group of DOPTZ-C3, successfully turning weak RTP emission into strong RTP emission.^69^

To maximize *k*_ISC_, great efforts have been devoted to narrowing Δ*E*_ST_. Traditional aromatic compounds with strong π-conjugation generally possess large exchange energy between S_1_ and T_1_ states, resulting in large Δ*E*_ST_. The effective pathway for narrowing Δ*E*_ST_ is introducing a donor–acceptor structure to generate an ICT state, which can effectively lower the energy gap between S_1_ and T_1_ states.^70^

Besides *k*_ISC_, accelerating the phosphorescence decay rate can effectively increase the EUE of T_1_ excitons and enhance RTP emission. As evaluated from the Einstein coefficient,^71^*k*_P_ is related to the transition dipole moment between T_1_ and S_0_ states:
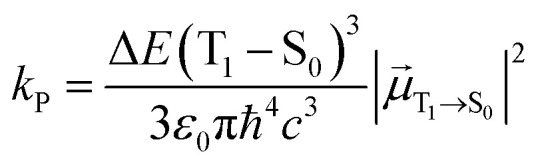


The transition dipole moment 
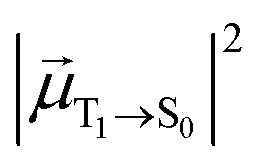
 can be expressed as:
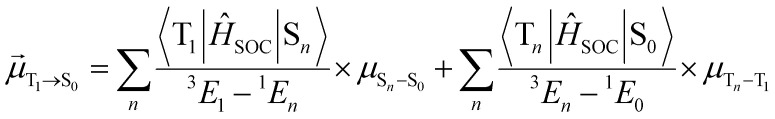


This expression underscores that the SOC matrix constant between singlet–triplet state manifolds is significant for *k*_P_. Meanwhile, increasing the transition dipole moment between S_*n*_ and S_0_ states can also facilitate *k*_P_, as revealed by Hirata.^72^

Overall, the realization of efficient RTP in purely organic systems is a multifaceted challenge that requires the synergistic optimization of both intrinsic molecular electronic structures and extrinsic environmental factors. From a photophysical perspective, enhancing SOC and minimizing Δ*E*_ST_ remain fundamental strategies to facilitate spin-flip processes, thereby effectively populating and utilizing triplet excitons. Simultaneously, the inherent sensitivity of triplet excitons to oxygen quenching and vibrational deactivation necessitates the meticulous engineering of the host matrix or crystalline packing. By providing a rigid microenvironment and modulating intermolecular interactions, these morphological strategies effectively suppress competitive non-radiative decay channels. Ultimately, a strategic balance between accelerating *k*_P_ and inhibiting quenching processes is the baseline for achieving RTP materials with both high quantum yields and long-lived emission, providing a robust foundation for their application in next-generation optoelectronic devices.

### Electroluminescence of RTP emitters

#### Mechanism of OLEDs

External quantum efficiency (EQE) is a pivotal parameter for evaluating the EL performance of OLEDs, representing the generation efficiency of electrically excited photons. EQE is expressed as:^73^EQE = *η*_e_*η*_r_*χΦ*_PL_ = *η*_e_ × IQEwhere *η*_e_ is the out-coupling efficiency of an OLED device, *η*_r_ represents the recombination efficiency of injected carriers, *χ* is the fraction of radiative excitons, and *Φ*_PL_ is the PL quantum yield of the emitter. For an emitter used in OLEDs, *Φ*_PL_ is a crucial factor that determines the theoretical upper limit of the EQE. According to spin statistics, electrically generated excitons consist 25% of singlets and 75% of triplets. The 75% triplet excitons in conventional fluorescence emitters are typically dissipated *via* non-radiative decay pathways, resulting in an EQE limited to below 5%. For emitters with near unity *Φ*_PL_, the EQE could reach 25–30%, in the absence of light-extraction technology. Generally, the efficiency of an OLED device will suffer from a roll-off as the current density increases due to the accumulation of excitons, as the high-concentration excitons will be consumed by non-radiative processes, such as TTA,^47^ STA,^74^ and triplet-polaron annihilation (TPA).^75^ Thus, controlling the exciton lifetimes is a critical method to alleviate exciton loss during the EL process. For RTP materials, the inherently long exciton lifetimes of T_1_ states are detrimental for OLED applications, thus a high *k*_P_ is preferable for electroluminescent RTP emitters.

Furthermore, sensitization technology, which aims to combine the excellent color purity of narrow-spectrum MR-TADF emitters with the high EUEs of sensitizers, has emerged as a pivotal strategy in high-definition displays.^76,77^ Förster resonance energy transfer (FRET) is the key mechanism affecting sensitization technology, which can be expressed as:^78^
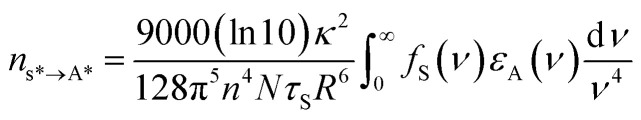
where *κ* is the orientation factor, *n* is the refractive index of the environment, *N* is the Avogadro constant, *τ*_S_ is the prompt decay lifetime of the sensitizer, and 
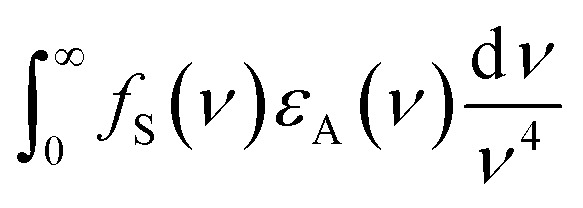
 is the overlap integral between the function of the sensitizer's spectrum and absorption of the dopant's spectrum. Based on this equation, an ideal sensitizer should possess a high radiative decay rate and an emission spectrum that sufficiently overlaps with the absorption spectrum of the dopant.

**Fig. 4:**
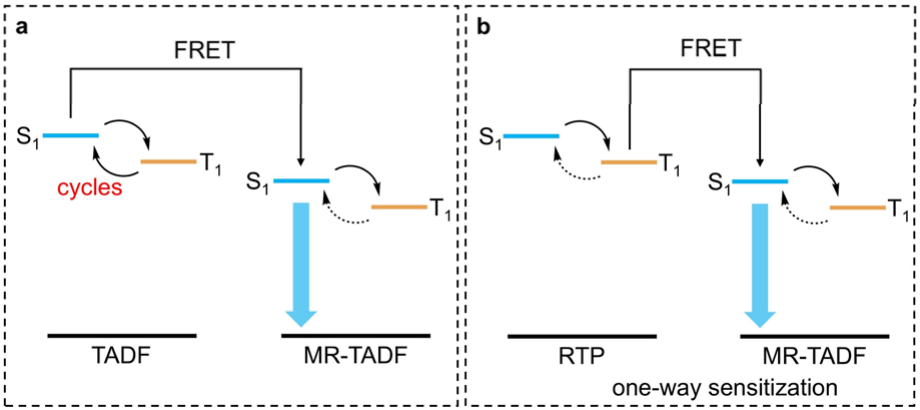
Energy transfer mechanisms of (a) TADF and (b) RTP sensitizers.

Although TADF sensitizers can utilize 100% of excitons in principle, the circulation between T_1_ and S_1_ states may cause long exciton lifetimes and a high probability of non-radiative decay, as illustrated in Fig. 4a. A radiative T_1_ state in an RTP emitter can effectively alleviate this problem, where the one-way process during sensitization can consume the excitons rapidly (Fig. 4b).^35,79^ During the FRET process from the T_1_ state to the S_1_ state, the strong SOC between the T_1_ and S_0_ states of RTP molecules provides the driving force for this spin-flip process of triplet excitons. Thus, purely organic RTP emitters have high potential as low-cost and high-efficiency sensitizers for use in OLEDs.

#### Conventional RTP emitters

**Fig. 5:**
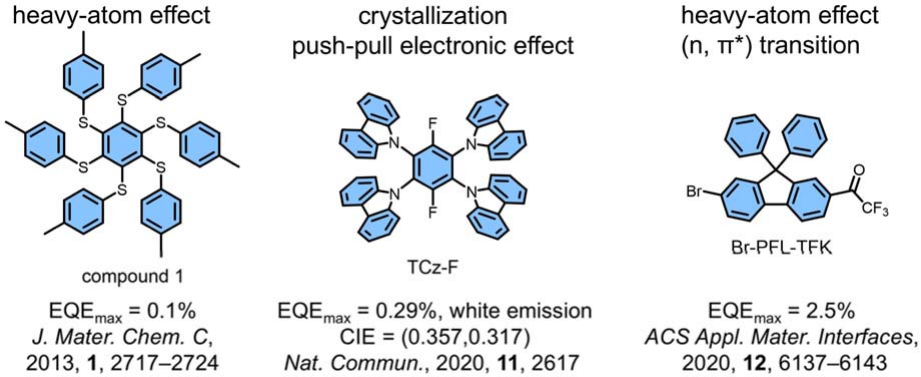
Molecular structures, mechanisms and corresponding EL performances of conventional RTP emitters.

The EL of RTP materials has been extensively explored for a long time. Conventional RTP materials are based on simple aromatic compounds with different decorated functional groups, wherein the film morphology and host matrix are significant for phosphorescence emission. One of the earliest examples of an RTP-based OLED was reported by Ceroni *et al.*, who synthesized compound 1, which consisted of a hexathiobenzene core and peripheral tolyl substituents (Fig. 5).^80^ The incorporation of sulfur atoms into compound 1 induced the heavy-atom effect, thereby facilitating the ISC process, and resulted in prominent phosphorescence emission. Although compound 1 possessed a high *Φ*_PL_ (80%) in powder, its doped film in a polymer host exhibited a significantly low *Φ*_PL_ (2%). A solution-processed OLED based on compound 1 yielded a maximum EQE (EQE_max_) of 0.1%, indicating poor EUE. Tang *et al.* synthesized a series of tetra-carbazole phenyl derivatives, TCz–F, TCz–H and TCz–OH, with different substitutions, exhibiting prominent RTP emission with long lifetimes of ∼2 s.^81^ EL in amorphous films displayed the dual-emission characteristics of fluorescence and phosphorescence. The optimal EL performance was achieved by TCz–F, which exhibited an EQE_max_ of 0.29% with Commission Internationale de l'Eclairage (CIE) coordinates of (0.357, 0.317), falling within the white emission region. Kim *et al.* functionalized spiro-fluorene with bromine and carbonyl fluoride to induce the heavy-atom effect and (n, π*) transitions, respectively, resulting in the RTP molecule BrPFL-TFK.^82^ BrPFL-TFK exhibited apparent phosphorescence emission in a PMMA matrix, with *Φ*_PL_ of 21.2% and *τ*_P_ of 5.28 ms. BrPFL-TFK was doped into three different hosts, CBP, mCP and PPT, for OLED fabrication. BrPFL-TFK displayed dual emission in CBP and mCP hosts, consisting of fluorescence and phosphorescence simultaneously, and pure phosphorescence emission was realized only in the PPT host. The device employing the PPT host radiated green phosphorescence emission with an EQE_max_ of 2.5%.

These examples have successfully applied RTP materials in OLEDs; however, the stringent requirements for host selection and device architecture limit their overall EL efficiencies. To compete with other high-efficiency sensitizers such as TADF and organometallic phosphorescence, breaking the limitations of EUE and host selection is critical for RTP emitters.

#### D–A-type RTP emitters and sensitizers

After the rapid development of TADF materials, the construction of ICT has emerged as a vital strategy for modulating excited states. ICT effectively reduces the exchange energy between S_1_ and T_1_ states, resulting in small Δ*E*_ST_s. The general approach for ICT involves adopting D–A structures into molecular skeletons, which can separate the highest occupied molecular orbital (HOMO) and the lowest unoccupied molecular orbital (LUMO). The separation of the spatial distributions of the HOMO and LUMO can also modulate SOC between triplet and singlet states, accelerating both ISC and *k*_P_. For such kinds of D–A-type RTP emitters, the host dependence is weakened as compared to those conventional RTP emitters lacking ICT characteristics, and the main considerations concern the matching of the triplet energy level and carrier transportability. Generally, carbazole-based host materials are preferred for these D–A-type RTP emitters, due to their high T_1_ energy levels and good carrier transportability.^83^ Additionally, phosphine-oxide-based hosts featuring high T_1_ energy levels and sterically crowded structures have also been applied to RTP emitters, as they can effectively restrict molecular motion *via* intermolecular hydrogen bonds and mitigate intermolecular interactions.^84^ The EL performances of representative RTP-based OLEDs and RTP-sensitized OLEDs are summarized in Tables 1 and 2.

**Table 1:** EL performances of RTP-based OLEDs^a^

Emitter	*τ*_P_	*Φ*_PL_ (%)	*λ*_EL_ (nm)	EQE_max_ (%)	Ref.
Compound 1	3.0 µs	80	485	0.1	80
TCz-F	520 ms	0.8	420, 580	0.29	81
Br-PFL-TFK	23.8 ms	24.0	∼518	2.5	82
DPTZN	87 µs	38	∼570	11.5	85
PSe1	0.61 ms	0.33	523	10.7	86
PSe2	0.64 ms	0.35	523	10.0	86
PSe3	0.93 ms	0.27	523	8.1	86
P(DMPAc-O-TPTrz)	1642.6 ns	49.5	495	9.7	87
P(DMPAc-O-TPOD)	12.67 ms	16.5	510	3.7	89
D32	763.0 ns	29.2	504	6.7	90
bTEoCN	15.2 ms	37.0	590	8.7	93
bTENCO	12.9 ms	68.0	∼540	18.9	93
bTEpCN	—	99.9	∼548	25.1	93
PXSeDRZ	1.21 ms	68.1	550	19.5	91
BPXSeDRZ	2.1 ms	66.3	554	17.2	92
DBPXSeDRZ	1.7 ms	66.9	567	17.9	92
XT-DPA	—	46.0	456	6.6	99
XT-*t*DPA	—	56.0	470	18.6	99
TXT-DPA	450.3 µs	72.0	474	17.7	99
TXT-*t*DPA	126.4 µs	93.0	486	27.3	99
2,3-PICz-XT	8.1 µs	92.0	508	32.7	100
3,2-PICz-XT	2.9 µs	72.0	522	24.9	101
3,2-PICz-TXT	1.4 µs	97.0	508	33.2	36

a Abbreviations: *τ*_P_ = phosphorescence lifetime of emitter in film; *Φ*_PL_ = photoluminescence quantum yield of emitter in film; *λ*_EL_ = EL peak of the OLED; EQE_max_ = maximum external quantum efficiency of the OLED.

**Table 2:** EL performances of RTP-sensitized OLEDs^a^

Sensitizer	Dopant	*λ*_EL_ (nm)	FWHM (nm)	CIE (*x*, *y*)	EQE_max_ (%)	Ref.
P(DMPAc-O-TPTrz)	TBRB^b^	—	—	(0.30, 0.43)	7.4	86
D32	S-Cz-BN^c^	490	18	(0.20, 0.42)	17.11	88
DBPXSeDRZ	DBP^c^	610	∼23	(0.61, 0.38)	10.1	90
2,3-PICz-XT	BN2^c^	540	42	(0.314, 0.639)	35.02	98
	BN3^c^	564	41	(0.439, 0.539)	35.13	
	PO-01-TB	556	—	(0.474, 0.513)	37.26	
	4CzTPNBu	556	—	(0.454, 0.515)	31.84	
	Ir(MDQ)_2_acac	598	—	(0.554, 0.403)	29.65	
	Ir(piq)_2_acac	620	—	(0.643, 0.330)	26.81	
	TPA-AQ	594	—	(0.478, 0.426)	30.10	
3,2-PICz-TXT	BN2^c^	540	40	(0.32, 0.65)	43.8	36
	tCzphB-Fl^c^	538	31	(0.29, 0.68)	43.8	
	tCzphB-Ph^c^	526	30	(0.23, 0.71)	40.9	

a Abbreviations: *λ*_EL_ = EL peak of the OLED; FWHM = full width at half-maximum of the spectrum; CIE = Commission Internationale de l'Eclairage coordinates; EQE_max_ = maximum external quantum efficiency of the OLED.

b The resulting device is a white-emission OLED, where the spectrum is composed of multiple peaks.

c These dopants feature narrow-spectrum emission.

Recently, Wang *et al.* integrated a sulfur-containing phenothiazine donor with a naphthalene core, realizing the orange-emitting RTP molecule DPTZN.^85^ Theoretical calculations revealed that DPTZN possessed two isomeric configurations. *Cis*-DPTZN, whose natural transition orbital (NTO) of T_1_ → S_0_ featured CT characteristics, possessed a large SOC matrix constant of 1.28 cm^−1^, while *trans*-DPTZN, featuring locally excited characteristics, exhibited a nearly zero SOC matrix constant. By doping DPTZN into a TRZ-BIM host, a good EQE_max_ of 11.5% was realized, with CIE coordinates of (0.46, 0.52). Lee *et al.* designed RTP materials *via* a similar strategy by selecting phenoselenazine containing a selenium atom as the electron donor.^86^ Three molecules, PSe1, PSe2 and PSe3, were synthesized where phenoselenazine was connected to a phenyl or biphenyl group in different positions. The EQE_max_s of PSe1, PSe2 and PSe3 were 10.7%, 10.0% and 8.1%, respectively, where the high exciton utilization could be attributed to the heavy-atom effect of selenium.

In 2020, Ding *et al.* designed the RTP polymer P(DMPAc-O-TPTrz) where the monomer was constructed with a D–O–A structure.^87^ The oxygen bridge could weaken the overlap between the HOMO and LUMO, suppressing CT fluorescence. The oxygen bridge also endowed the monomer with enhanced SOC, which facilitated the spin-flip process from a singlet to a triplet state. The resulting polymer-based OLED displayed a high EQE_max_ of 9.7%. In addition, P(DMPAc-O-TPTrz) was also used as a sensitizer for the orange emitter TBRB, yielding white emission with EQE_max_ of 7.4% and CIE coordinates of (0.30, 0.43).^88^ Inspired by this phenomenon, Ding *et al.* substituted a triazine acceptor with dibenzothiophene-S,S-dioxide,^89^ realizing the white-emission polymer P(DMPAc-O-DBTDO). By adjusting the doping concentration of P(DMPAc-O-DBTDO), correlated color temperatures could range from 4834 to 6741 K, with a high color rendering index above 65. EQE_max_ of 3.7% was obtained at a doping concentration of 20 wt%, with Commission Internationale de l’Eclairage (CIE) coordinates of (0.30, 0.44). A benzophenone acceptor was further introduced to this D–O–A structure, and the small-molecule RTP emitter D32 was obtained.^90^ D32 possessed EQE_max_ of 6.70% in an OLED, exhibiting cyan phosphorescence. D32 was also utilized as a sensitizer for the MR-TADF emitter S-CZ-BN, realizing narrow-spectrum emission peaking at 490 nm, with EQE_max_ of 17.11%.

**Fig. 6:**
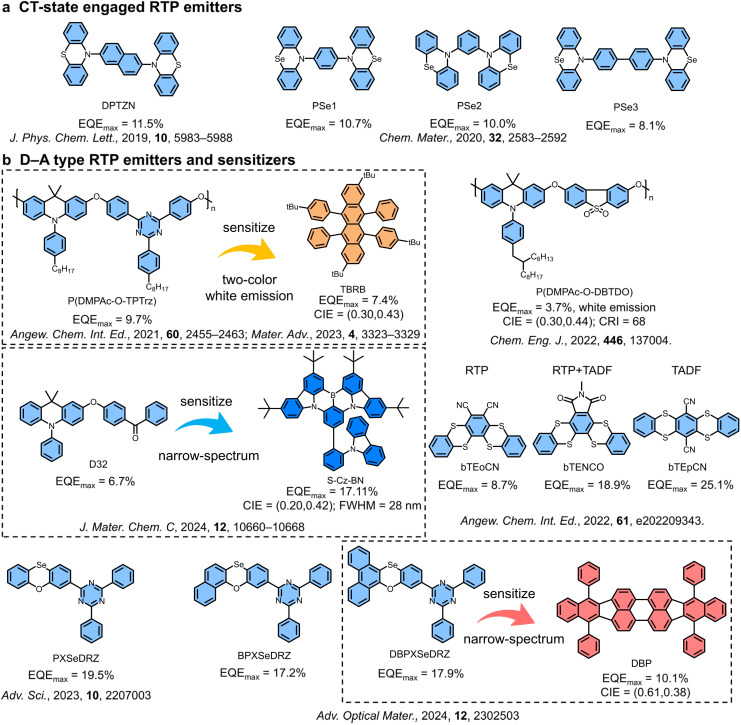
Molecular structures and corresponding EL performances of (a) CT-state-engaged and (b) D–A-type RTP emitters and sensitizers. The molecules inside boxes represent pairs of sensitizers and dopants.

Su *et al.* also contributed numerous high-efficiency RTP materials for OLEDs. The selenium-containing electron donor phenoxaselenine was connected to three different acceptors, 2DPm, 4DPm and DRZ, resulting in three D–A-type molecules: PXSe2DPm, PXSe4DPm and PXSe2DRZ.^91^ The DRZ acceptor established intramolecular confinement through hydrogen bonds and also endowed PXSe with a folded configuration. The lone-pair electrons in the selenium atom were not parallel with the orbitals on the DRZ acceptor, providing excess angular momentum change for the spin-flipping of excitons. OLEDs based on PXSe2DPm, PXSe4DPm and PXSe2DRZ exhibited EQE_max_s of 7.3%, 16.0% and 19.5%, respectively. PXSe2DRZ demonstrated pure phosphorescence emission and comparable EL efficiencies to those of metallic phosphors. By extending PXSe with naphthalene and phenanthrene, which contain large-scale π-conjugation, the resulting molecules BPXSeDRZ and DBPSeDRZ possessed red-shifted RTP emission.^92^ BPXSeDRZ and DBPSeDRZ also performed well in OLEDs, where EQE_max_ reached 17.2% and 17.9%, respectively. Moreover, DBPSeDRZ was adopted as a sensitizer for a red conventional fluorescence emitter DBP, realizing EQE_max_ of 10.1% with CIE coordinates of (0.61, 0.38). In addition, rigid polycyclic RTP emitters were also developed by the same group.^93^ Sulfur-bridged molecular skeletons displayed different photophysical properties upon alternating the bridge position, where pure RTP, combined RTP and TADF, and pure TADF properties could be obtained through molecular engineering. The pentaphene-based molecule bTEoCN possessed a folded structure and exhibited pure RTP emission, while the pentacene-based molecule bTEpCN, with a symmetric structure, somehow showed TADF properties. bTENCO, which integrated an imide into the bTEoCN skeleton, displayed dual emission containing RTP and TADF (Fig. 6b). OLEDs based on bTEoCN, bTENCO and bTEpCN showed EQE_max_s of 8.7%, 18.9% and 25.1%, respectively. The spectrum of bTEoCN was composed of both fluorescence and phosphorescence, while pure TADF emission was observed for bTEpCN. This work discovered the RTP phenomenon in polycyclic aromatic rings, bringing about new guidance for realizing high-efficiency purely organic phosphors.

#### Carbonyl-based RTP emitters and sensitizers

Although RTP-based OLEDs have been extensively researched, their application as purely organic sensitizers is a quite recent development. The relatively low efficiencies of early developed RTP materials made them inappropriate as sensitizers. Moreover, some RTP molecules containing halogen or hydroxy groups may have a negative effect on device stability.

The carbonyl group is a classical electron acceptor characterized by lone-pair electrons, which can facilitate (n, π*) transitions in organic molecules. Several D–A-type TADF emitters containing carbonyl groups have been developed, which can realize high efficiency and impressive stability. In the past few years, we have comprehensively studied organic luminescent materials based on carbonyl-containing acceptors^19,32,64,94–98^ and found that the distinct (n, π*) transition enhances SOC between the singlet and triplet manifolds, while concentration quenching is suppressed by the large steric hindrance of carbonyl acceptors (benzophenone, xanthone, acridone, *etc.*). Very recently, we discovered RTP phenomena in carbonyl-based emitters, establishing a new set of robust sensitizers.

**Fig. 7:**
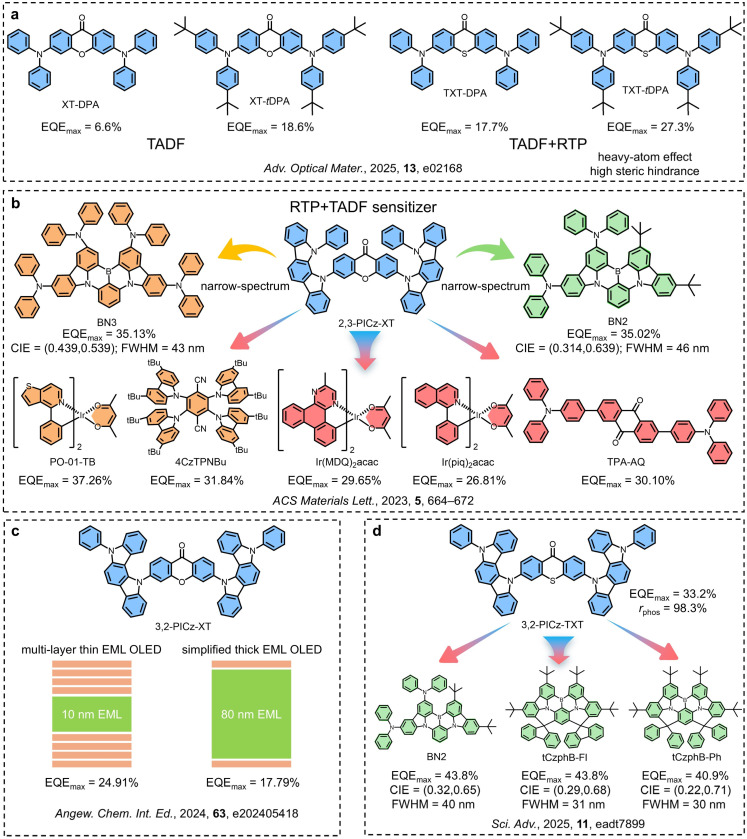
(a) Molecular structures and EL efficiencies of the TADF and RTP emitters XT-DPA, XT-*t*DPA, TXT-DPA and TXT-*t*DPA. (b) A summary of the RTP and TADF dual-emission sensitizer 2,3-PICz-XT and dopants. BN2 and BN3 are narrow-spectrum MR-TADF emitters; PO-01-TB, Ir(MDQ)_2_acac and Ir(piq)_2_acac are metallic phosphorescence emitters; and 4CzTPNBu and TPA-AQ are TADF emitters. (c) A summary of the RTP emitter 3,2-PICz-XT; the OLEDs are based on non-doped films. (d) A summary of the RTP sensitizer 3,2-PICz-TXT. BN2, tCzphB-Fl and tCzphB-Ph are narrow-spectrum MR-TADF emitters.

As discussed, the vibrational deactivation of triplet excitons can be reduced by rigidifying molecular skeletons, facilitating RTP emission. Two flexible electron donors, diphenylamine (DPA) and *tert*-butyl-diphenylamine (*t*DPA), are introduced into the carbonyl-based electron acceptor xanthone (XT) or thioxanthone (TXT), generating four luminescent molecules: XT-DPA, XT-*t*DPA, TXT-DPA and TXT-*t*DPA (Fig. 7a).^99^ The XT-based molecules XT-DPA and XT-*t*DPA displayed TADF emission but no phosphorescence emission at room temperature. In contrast, TXT-DPA and TXT-*t*DPA exhibited dual-channel emission of TADF and RTP, owing to the heavy-atom effect from the sulfur atom in TXT. Doped OLEDs based on XT-DPA and XT-*t*DPA possessed EQE_max_s of 6.6% and 18.6%, respectively, inferior to TXT-DPA (17.7%) and TXT-*t*DPA (27.3%). The EL differences between XT and TXT emitters demonstrate the critical role of the heavy-atom effect, and the presence of a phosphorescence decay path could increase the EUEs of OLEDs.

Indolocarbazole isomers with a rigid skeleton and large π-conjugated planes are widely used electron donors. Two 11-phenylindolo[2,3-*a*]carbazole (2,3-PICz) molecules connected to an XT acceptor resulted in the D–A–D-type molecule 2,3-PICz-XT, which can open up a radiative path for triplet excitons.^100^ Large SOC matrix constants between S_1_ and T_1_, T_2_, and T_3_ states were confirmed by theoretical calculations, which contributed to the engagement of (n, π*) transition. The phosphorescence was confirmed by bubbling oxygen into toluene solutions, where a prominent blue-shifted and weakened emission spectrum was observed. The PL spectrum measured with a 1-ms delay also exhibited pronounced red shifts. Based on a comprehensive analysis of experimental data, it was confirmed that 2,3-PICz-XT showed the dual-emission characteristics of both fluorescence and phosphorescence. OLEDs employing 2,3-PICz-XT as an emitter exhibited the highest EQE_max_ of 32.73% at a doping concentration of 20 wt% in a PPF host, peaking at 508 nm. Furthermore, 2,3-PICz-XT was adopted as a sensitizer for a metallic phosphorescence emitter, TADF emitter and MR-TADF emitter, exhibiting impressive EQE_max_s ranging from 26.81% to 37.26% (Fig. 7b). Notably, the red emitters Ir(MDQ)_2_acac and TPA-AQ achieved remarkably high EQE_max_s of 29.65% and 30.10%, respectively, representing some of the highest EL efficiencies reported for these emitters. The core mechanism is that the delayed fluorescence and phosphorescence can sensitize dopants through FRET simultaneously, realizing multi-channel sensitization.

Through the isomeric strategy of an electron donor, 3,2-PICz-XT was synthesized, which possessed a phosphorescence quantum yield (*Φ*_P_) and fluorescence quantum yield (*Φ*_F_) of 30% and 42%, respectively (Fig. 7c).^101^*k*_P_ of 3,2-PICz-XT was 1.03 × 10^5^ s^−1^, remarkably faster than those of generally reported purely organic RTP molecules. Non-doped OLEDs based on 3,2-PICz-XT displayed a high EQE_max_ of 24.91%, with negligible roll-off at 1000 cd m^−2^.3,2-PICz-XT was further applied in a simplified ultra-thick OLED, where the EML was 80 nm, and a high EQE_max_ of 17.79% was obtained. The non-doped OLED based on 3,2-PICz-XT is prominently better than those based on carbonyl-based RTP emitters with DPA or *t*DPA donors. Such improvement of non-doped OLEDs is mainly attributed to the rigidified molecular structures, which stabilized triplet excitons and facilitated carrier transport in the amorphous film.

To further increase the SOC matrix constants, 3,2-PICz-TXT with a TXT acceptor was designed and synthesized (Fig. 7d).^36^ The purely organic RTP emitter 3,2-PICz-TXT owned a high phosphorescence ratio of 98.3%, with a fast *k*_P_ of 6.8 × 10^5^ s^−1^. An OLED utilizing 3,2-PICz-TXT as an emitter showed an outstanding EQE_max_ of 33.2%, which exceeded the commercial metallic phosphorescent emitter Ir(ppy)_3_ (25.2%) in the same device structure. By employing 3,2-PICz-TXT as a sensitizer, the three MR-TADF dopants BN2, tCzphB-Fl and tCzphB-Ph realized ultrahigh EQE_max_s of 43.8%, 43.3% and 40.9%, respectively. More impressively, the power efficiencies of the devices based on BN2 and tCzphB-Fl were even close to 200 lm W^−1^. These devices represent state-of-the-art narrow-spectrum OLEDs. In addition, the operational lifetime can also be enhanced by this RTP sensitizer, and the LT_80_ lifetime (the time when the luminance decays to 80% of the initial luminance) of tCzphB-Fl can be raised from 15.0 to 110.1 h under sensitization with 3,2-PICz-TXT.

Notably, the horizontal dipole ratios of 2,3-PICz-XT and 3,2-PICz-XT are as high as 82.7% and 78.0%, respectively. The D–A structure can effectively enhance intramolecular polarity, facilitating orientation polarization.^102^ Meanwhile, the rigid and highly planar PICz donors expand the molecular planes where the transition dipole moment lies. These carbonyl-based RTP emitters are instructive examples for designing horizontally oriented molecules.^103^

## Conclusions and outlook

Significant efforts have been devoted to the molecular design and OLED applications of RTP emitters and sensitizers. In this perspective, the design principles of RTP emitters, which have been thoroughly explored in the past few decades, are briefly summarized. More importantly, the significance of developing RTP sensitizers is emphasized, as they are pivotal for next-generation OLED displays. The one-way decay characteristics of RTP sensitizers can resolve exciton cycling issues, thereby achieving high efficiency and stability. The D–A skeleton is an ideal molecular design strategy for high-efficiency RTP sensitizers, where the incorporation of lone-pair electrons and heavy atoms is also necessary. Practically applicable RTP sensitizers based on carbonyl-containing XT and TXT acceptors can be prospectively developed through synergistic molecular design strategies.

The core issue concerning RTP sensitizers remains their lack of stability. To alleviate triplet exciton quenching, the rate constants *k*_ISC_ and *k*_P_ should be further increased, which would reduce the lifetimes of triplet excitons. Meanwhile, suitable heavy atoms should be carefully selected during molecular design, since their introduction may weaken the chemical bonding energies. In addition, efficient blue RTP emitters are still rare. The realization of blue emission from organic emitters is highly dependent on shortening the π-conjugation length or weakening ICT effects, which restricts the structural multiplicity of emitters. Although a few studies have successfully developed blue RTP emitters,^104–106^ their reliance on specific rigid polymer host matrices limits their practical application in OLEDs. In that case, the development of high-performance blue RTP-OLEDs still demands substantial research effort. Furthermore, compatible functional materials (*e.g.* host and blocking materials) should be further developed to maximize the performance of RTP materials.

## Author contributions

Z. Zhao conceived the project. H. Liu and Y. Fu organized and wrote the paper. Z. Zhao revised the paper. Z. Zhao, B. Z. Tang and J. W. Y. Lam supervised the project.

## Conflicts of interest

The authors declare no conflicts of interest.

## Data Availability

Data sharing is not applicable – no new data was generated.
